# Gut Microbiota Metabolites in Major Depressive Disorder—Deep Insights into Their Pathophysiological Role and Potential Translational Applications

**DOI:** 10.3390/metabo12010050

**Published:** 2022-01-08

**Authors:** Miguel A. Ortega, Miguel Angel Alvarez-Mon, Cielo García-Montero, Oscar Fraile-Martinez, Luis G. Guijarro, Guillermo Lahera, Jorge Monserrat, Paula Valls, Fernando Mora, Roberto Rodríguez-Jiménez, Javier Quintero, Melchor Álvarez-Mon

**Affiliations:** 1 Department of Medicine and Medical Specialities, University of Alcala, 28801 Alcalá de Henares, Spain; miguel.angel.ortega92@gmail.com (M.A.O.); cielo.gmontero@gmail.com (C.G.-M.); oscarfra.7@hotmail.com (O.F.-M.); guillermo.lahera@gmail.com (G.L.); jorge.monserrat@uah.es (J.M.); paolavallsp@gmail.com (P.V.); mademons@gmail.com (M.Á.-M.); 2 Ramón y Cajal Institute of Sanitary Research (IRYCIS), 28034 Madrid, Spain; luis.gonzalez@uah.es; 3 Cancer Registry and Pathology Department, Hospital Universitario Principe de Asturias, 28806 Alcalá de Henares, Spain; 4 Department of Psychiatry and Mental Health, Hospital Universitario Infanta Leonor, 28031 Madrid, Spain; fernando.mora@salud.madrid.org (F.M.); fjquinterog@salud.madrid.org (J.Q.); 5 Unit of Biochemistry and Molecular Biology (CIBEREHD), Department of System Biology, University of Alcalá, 28801 Alcalá de Henares, Spain; 6 Psychiatry Service, Center for Biomedical Research in the Mental Health Network, University Hospital Príncipe de Asturias, 28806 Alcalá de Henares, Spain; 7 Department of Legal Medicine and Psychiatry, Complutense University, 28040 Madrid, Spain; rodriguez.jimenez.psiquiatra@gmail.com; 8 Institute for Health Research 12 de Octubre Hospital, (Imas 12)/CIBERSAM (Biomedical Research Networking Centre in Mental Health), 28041 Madrid, Spain; 9 Immune System Diseases-Rheumatology, Oncology Service an Internal Medicine, University Hospital Príncipe de Asturias, (CIBEREHD), 28806 Alcalá de Henares, Spain

**Keywords:** gut microbiota, dysbiosis, microbiota-gut-brain axis, microbial metabolites, malnutrition, short-chain fatty acids, neurotransmitter

## Abstract

The gut microbiota is a complex and dynamic ecosystem essential for the proper functioning of the organism, affecting the health and disease status of the individuals. There is continuous and bidirectional communication between gut microbiota and the host, conforming to a unique entity known as “holobiont”. Among these crosstalk mechanisms, the gut microbiota synthesizes a broad spectrum of bioactive compounds or metabolites which exert pleiotropic effects on the human organism. Many of these microbial metabolites can cross the blood–brain barrier (BBB) or have significant effects on the brain, playing a key role in the so-called microbiota-gut-brain axis. An altered microbiota-gut-brain (MGB) axis is a major characteristic of many neuropsychiatric disorders, including major depressive disorder (MDD). Significative differences between gut eubiosis and dysbiosis in mental disorders like MDD with their different metabolite composition and concentrations are being discussed. In the present review, the main microbial metabolites (short-chain fatty acids -SCFAs-, bile acids, amino acids, tryptophan -trp- derivatives, and more), their signaling pathways and functions will be summarized to explain part of MDD pathophysiology. Conclusions from promising translational approaches related to microbial metabolome will be addressed in more depth to discuss their possible clinical value in the management of MDD patients.

## 1. Introduction

Microorganisms belonging to the human body are acquiring more relevance nowadays as they establish mutualistic relationships with our cells modulating our health status. Since the early 1990s, Lynn Margulis stated the basis of endosymbiotic theories and coined the term “holobiont”, explaining the coevolution of symbionts and host [1], microbes inhabiting the human being have gained more attention in health research and clinical practice. Although today we also know that nearly 50 years before, Adolf Meyer-Abich had already started talking about his forgotten and not spread theory of “holobiosis” concept defending that the assemblage of independent organisms could create a strong whole “holobiome” [2]. There are different ecosystems of bacteria, protozoa, archaea, and viruses distributed along the whole body (skin, mouth, intestine, vagina…) playing a key role in the homeostasis by communicating bidirectionally with host immune cells and functions. In this context, the Human Microbiome Project (HMP) was born in 2007 collecting biological and clinical data for the research of microbiome populations and behavior in health and disease [3]. The scientific bibliography alleges that the correct proportion of microbes with respect to human cells is approximately 1:1, being bacteria the most representative kingdom [4].

Gut microbiota is considered as a dynamic organ, as some say, “the last human organ under active research” [5]. Its microbial composition varies across the digestive tract, being the colon, the place containing the largest population of bacteria in the organism (3.8 × 10^13^) with the dominant phyla: Firmicutes and Bacteroidetes in a proportion of 90%, and a 10% of Actinobacteria, Proteobacteria, Fusobacteria, and Verrucomicrobia [6]. Diversity and relative abundances of different species of bacteria also vary through the individual’s life stages, being some beneficial genera declined with associated age progression [7]. The dynamism of gut microbes is heavily shaped by environmental factors, being the diet the greatest but not the only one. All these vital factors that alter gut microbiota homeostasis are also the essential pillars of health: diet, physical activity, stress, and sleep hygiene, all these affected by chronobiology.

Microbial dysbiosis can contribute to alterations in the physiological response [8], setting the progression of inflammatory and neurodegenerative diseases [9]. The impaired gut microbiome operation modulating the disrupted immunostasis makes the host being more susceptible to infections, hypersensitivity reactions, autoimmunity, chronic inflammation, and even cancer [10]. The dysbiosis condition is known to have a significant correlation with immunological and neuroimmunological status in physical and psychological pathologies. This imbalance leads to a disruption in the already known microbiota-gut-brain axis (MGB) and explains part of the altered microbial and human production of neurotransmitters like serotonin, dopamine, gamma-aminobutyric acid (GABA), and noradrenaline [11], and the distorted crosstalk dialogue of other microbial metabolites with neuroimmune functions. All in all, the health of the ecosystem that harbors the intestine, signifies one more key element in the complex network of multifactorial non-communicable diseases, many of them being comorbid metabolic diseases and mental disorders like the greater and greater emerging Major Depressive Disorder (MDD).

MDD is approximately affecting 350 million people in the world, which implies nearly 5% of the entire population and supposes the third leading cause of disability. The latest numbers collected by the Global Health Data Exchange (GHDx) and the World Health Organization (WHO) show that in its most severe manifestation, this incapacitating condition leads to 700,000 suicide commitments every year, ranking the fourth cause of death among individuals in the 15–19 age group; and it is also estimated that more than 3 out of 4 patients do not ask for help and not receive treatments in low and middle-income countries, where perhaps social stigma is greater [12,13]. There are differences of prevalence regarding sex too: more women suffer from depression than men [14] and more men die due to suicide than women [15].

Healthcare costs and economic burden derived from the loss of productivity reach more than 200 billion $. The quantity is expected to keep rising these days due to the impact of COVID-19 pandemic times, which has exacerbated more social stressors like isolation and unemployment besides the illness [16]. At the same time, this public health emergency, considered also as a global psychological pandemic [17], has promoted to invest in finding mental wellbeing in most countries [18], which hopefully has shed more light to mitigate the barriers of self and public’s beliefs and stigma related to mental health disorders.

Every time, it is becoming more evident that the rising prevalence of depression, especially in developed countries, is linked to changes in dietary and sleep patterns derived from high levels of stress related to occupations, studies, and social relationships, among others. The research suggests that the base medical treatment frequently tends to fail when those aspects are not addressed [19] as psychiatric drug therapies alone, although may stabilize the patient, are not enough to cure the disorder. For this reason, nowadays holistic approaches are kept in mind for improving mental health care. This management may include nutritional intervention, peer supports, self-control of stress with psychotherapy and guided mindfulness techniques; and, encouragement for practicing physical exercise and re-introducing good habits to ameliorate the individual’s chronobiology, inflammatory status, and symptoms.

In the present work, we will focus on the evidence from adjuvant therapies primarily based on nutritional intervention with supplementation or advising certain foods containing prebiotics that boost the metabolome function in the so appealing MGB axis. After brief statements about the biological basis of MDD and microbial metabolites motion in the pathophysiology of this condition; this descriptive review mainly aims to gather the most recent updates related to translational applications of bioactive compounds equivalent to microbial products and other feasible ways to target gut microbiota functioning, from research to clinical practice, all in the MDD framework.

## 2. The Role of Gut Microbiota in Major Depression: The Microbiota-Gut-Brain Axis

Once upon a time, the brain was thought to be an organ with relative independence from the rest of the body. Nowadays it is widely accepted that there is an intricate, bidirectional, and integrative brain-body interplay which may be summarized by the famous quote “*Mens sana in corpore sano*” [20,21,22]. In this sense, different systems have been recognized in this relationship, like the psychoneuroimmunoendocrinology [23] or the interaction between the psychology, nervous, endocrine, and immune system-, a heart–brain communication [24] or how the brain affects and is affected by the heart- and of course a muscle–brain crosstalk [25] the regulation of muscle contraction from the nervous system and their derived effects on the proper brain. One structure should perhaps be noticed as a critical modulator of proper brain functioning and well-being, the gut. Indeed, several authors frequently considered the gut as the “second brain” [26]. As the gut microbiota is a major representative element located in this organ with pleiotropic and vital functions, not only at local but also at systemic levels, the authors have designed an MGB axis to the complex relationship between these elements [27].

There are three main mechanisms by which the MGB axis works. The first is related to the presence of a prominent innervation in the gut, represented by an intrinsic or enteric nervous system (ENS) and an extrinsic or central nervous system (CNS). The former is essential for gastrointestinal functioning and the second is responsible for a thorough monitoring and regulation of this system through three different pathways: (1) The vagus nerve, projected from the brainstem; (2) The thoracolumbar spinal cord and (3) Lumbosacral spinal cord, all of them presenting afferent (sensory) innervation and efferent (motor innervation) [28]. It is of note that both the ENS and CNS fulfill mutual actions to control the gut activity and the microbiota. In turn, the microbiota and intestinal cells may also influence the neural networks of the ENS and CNS. For instance, the release of some neurotransmitters by the efferent neurons from the CNS in the gut may be collected by certain types of bacteria, determining the growth of those microbial populations and simultaneously, the presence of some microorganism may produce or modulate the release of some neurotransmitters and metabolites from the gut to the afferent pathways to the CNS [29]. Thus, through this mechanism, the brain may influence the gut and microbiota function while the microbiota and the gut may orchestrate the CNS and brain activity. The second great mechanism by which the MGB axis works is the immune system. There are several mechanisms implicated in the gut microbiota-immune systems interplay, including microbial products, inflammatory cytokines, and the proper bacterial metabolites [30]. An adequate microbial composition (eubiosis) is related to a proper and regulated immune response. Conversely, an altered microbiota (dysbiosis) is accompanied by an exacerbated immune response and damage in the intestinal barrier. Eventually, this may lead to bacterial translocation and endotoxemia, enhancing a condition of systemic inflammation, negatively affecting the brain [31,32,33]. Finally, the third pathway involved in the MGB axis is the systemic widespread to the bloodstream of several elements, including hormones, metabolites, or neurotransmitters [34].

**Figure 1:**
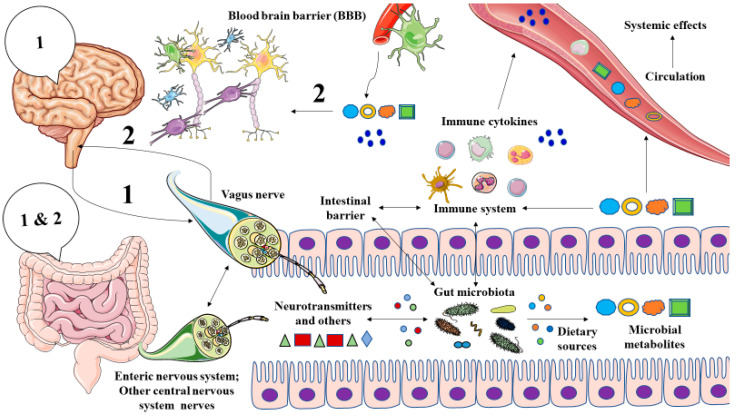
A general picture of the microbiota-gut-brain axis. As represented, there is bidirectional communication between the brain and the gut through different mechanisms. The vagus nerve may send signals (“1”) from the brain to the gut, although there may be other indirect mechanisms like endocrine factors that could reach the gut. The gut, and prominently, the microbiota integrates these signals and in turn, they respond by different pathways to the brain. Those mechanisms include (1) Vagal activation, due to the direct action of the gut microbiota or through the interactions with other nervous fibers from the enteric nervous system or central nervous system; (2) By influencing the local and systemic immune system and intestinal barrier, leading to the production of a set of signaling molecules -mainly cytokines- that are distributed via systemic circulation and (3) Thanks to the production of different microbial metabolites that are either transported by systemic circulation or exert immunomodulatory effects, or stimulate the vagus nerve or signaling in different tissues within the body. Both cytokines and microbial metabolites can cross the blood–brain barrier, therefore providing an effect on the brain. Overall, these 3 mechanisms send a signal to the brain (“2”), which integrates the information received from the gut.

As summarized in Figure 1, the gut microbiota is a major source of several metabolites with critical functions in the MGB axis by several pathways, including the stimulation of the vagus nerve, modulating the gut, systemic and neuroinflammation, central neurotransmission, and the integrity of the blood–brain barrier (BBB), also binding to host receptors in the brain [35,36]. Several studies have reported the role of abnormal metabolite production by gut microbiota in different neuropsychiatric disorders [37,38,39]. Thus, in this study, we will explore the role of microbial metabolites in the brain with a special focus on their role in the pathophysiology of MDD and the main translational opportunities derived from this knowledge.

## 3. Microbial Metabolites in MDD

### 3.1. Biological Basis of MDD

MDD is a multifactorial and entangling concern with many psychosocial and biological changes implicated. What causes MDD in an individual is not fully understood and several factors appear to be involved in the origin of this complex disorder. For instance, particular polymorphisms in some genes affecting the functioning of various critical areas in the brain appear to be associated with a higher risk of suffering from MDD [40,41]. However, no single gene has been identified as a clear causative agent of MDD and genetic susceptibility may be accompanied by other environmental factors to result in MDD. Early-life stress (ELS) is perhaps one of the most plausible causative agents related to the development of MDD and previous studies have demonstrated an increased risk of suffering from depression and many psychiatric disorders in individuals exposed to ELS, even in childhood and adolescence [42,43]. The biological explanation of this association is that ELS triggers an epigenetic reprogramming in the brain leading to the onset of several pathophysiological mechanisms involved in the genesis of MDD [44]. Similar effects may be attributed to the exposure to chronic stress, driving to the development of MDD [45]. Another pivotal determinant related to the increased risk of MDD that should be noticed here includes sex. Prior epidemiological studies have found that the risk of MDD is approximately twofold higher in women than men, although this difference may be even higher in younger ages [14]. Different reasons have been given here, including genetic vulnerability, hormonal fluctuations related to reproductive function, and a plethora of psychosocial factors [46]. This means that men and women often display different symptoms and behaviors in response to MDD [15]. Thus, sex should be considered as an important variable related to the development of MDD although many social and cultural determinants may also contribute to the pathophysiology of MDD [47].

The biological basis of the MDD could be classified at three great levels: (1) Molecular and cellular alterations affecting the neurons and glia; (2) Structural and functional impairment in key brain regions and (3) Systemic factors that hit the central nervous system. The molecular and cellular basis of MDD involves a set of alterations in the neuronal and glial behavior, function, communication, and even on their viability that are observed in depressed subjects. These changes often occur in certain vital regions of the brain which may increase or reduce their size as well as their connectivity and functionality [48]. Besides, systemic signals from the rest of the body may impact the brain directly or indirectly. Thus, a broad spectrum of mechanisms is working in the pathobiology of MDD as it will be summarized hereunder.

Within the brain, neurons are strongly dysregulated in depressed individuals. There are plenty of molecular changes in these cells, all related to a dysregulation of neuroprotective and neurotrophic signals. In this sense, it has been elucidated the central role of brain-derived neurotrophic factor (BDNF), a critical molecule downregulated in MDD with important effects in neurogenesis, neural growth, maturation, survival, and neuroplasticity [49]. Similar consequences are also observed for the glial cells, which are notably reduced in frontal cortical and limbic brain regions [50]. An important consequence of this glial dysregulation is a synaptic dysfunction in these areas that are considered as one of the major pathophysiological mechanisms of MDD [51]. The monoamine hypothesis was one of the first discoveries of disrupted neurotransmission in MDD. This theory supports that the monoamines (serotonin, dopamine, and noradrenaline) are importantly dysregulated in the brain of depressed patients and the evidence shows that many of the antidepressants target these components [52]. However, the role of monoamines in the pathophysiology of MDD is incomplete, as a representative group of the patients may only exert a partial or absent response to these therapies received thereby supporting that the monoamines are a part but not the entire picture of MDD [53,54]. Other important neurotransmitters dysregulated in MDD are GABA, acetylcholine, and glutamate.

Likewise, different neuropeptides are also implicated in the pathophysiology of MDD. Vasopressin (AVP) and oxytocin (OXT), two main nonapeptides prominently involved in a plethora of social and behavioral functions seem to be significantly affected in depressed subjects. It is thought that an imbalance between AVP and OXT in favor of the former may be related to the development of MDD [55]. AVP and OXT are also related to an abnormal functioning of the Hypothalamic Pituitary Adrenal (HPA) axis, a central mechanism in many psychiatric disorders like MDD [56]. The altered HPA axis is strongly related to the body’s response to stress. This system is frequently hyperactivated in depressed subjects, characterized by an augmented corticotropin release factor (CRF), reduced negative feedback probably due to a resistance of glucocorticoid receptors (GR) and hypercortisolism [57]. Melatonin, a major neuropeptide involved in circadian rhythms is equally an important point of study in many psychiatric disorders, as depressed patients show an altered melatonin action and chronobiology that may be considered in the development of effective therapies [58].

Oxidative stress (OS) is an important local and systemic marker of damage observed in patients with MDD [59]. OS is the result of an imbalance of oxidants (free radicals) production with regard to antioxidants, leading to local damage in the brain with systemic consequences [60]. Aberrant neuroinflammation is a major source of OS in the brain and turn, the OS is a key driver of the neuroinflammatory process [61]. Besides, compelling evidence has demonstrated that neuroinflammation is a key factor involved in the abnormal neurotransmission, HPA axis, and neurogenesis, with a pivotal role of the microglia in all these events [62]. The origin of the neuroinflammation may be either primary due to an impaired vascular function in the brain and/or underlying damage in this structure or secondary being related to any inflammatory condition related to an augmented cytokine production that is able to cross the BBB [63].

As above mentioned, a disrupted MGB axis is implicated in the development of several neuropsychiatric diseases including MDD, which has been proven to influence several pathophysiological mechanisms implicated in this mental disorder [64]. In the following section, we will summarize the role of microbial metabolites, a crucial way of communication in the MGB axis in the pathophysiology of MDD, with a special emphasis on the bidirectional implications of the brain and gut disturbances.

### 3.2. Microbial Metabolites Involved in the Pathophysiology of MDD

By using the different nutrients and components ingested in the diet, the gut microbiota produces different metabolites targeting the proper microbiota and host cells [65]. Technological advances in the field of metagenomics, metatranscriptomics, and metabolomics have facilitated a better comprehension of the gut microbiota functionality, having brought the discovery of plenty microbial metabolites with prominent consequences for health and disease [66]. Hereunder, we will describe some of the most relevant microbial metabolites involved in the development of MDD.

#### 3.2.1. Short-Chain Fatty Acids

Short-chain fatty acids (SCFAs) are perhaps one of the most deeply studied metabolites derived from gut microbiota. SCFAs are a subtype of fatty acids notably abundant in the proximal colon due to the fermentation of partial and non-digestible polysaccharides like dietary fiber or resistant starch [67]. There are three main types of SCFAs: Butyrate, propionate, and acetate. *Eubacterium rectale*, *Roseburia faecis*, *Eubacterium hallii*, and *Faecalibacterium prausnitzii* appear to be the major producers of butyrate in the gut [68]. *Veilonella* spp., *Lactobacillus* spp., *Bacteroides* spp., and *Propionibacterium* spp. are some of the most representative bacteria involved in propionate production [69]. Conversely, acetate production is commonly spread among numerous bacterial classes [70]. Virtually all cell types present at least one type of SCFA receptors, which include free fatty acid receptor-2 and -3 (FFAR2 and FFAR3), G-Protein-Coupled receptor (GPCR) like GPR43, GPR41, GPR109a, and Olfr78 [71,72].

The local actions of SCFAs in the gut are multiple, aiding to keep the intestinal integrity by regulating the luminal pH, mucus production, epithelial cells activity, and the immune system, being also crucial for microbial communication, affecting quorum sensing and limiting the invasion or growth of different microorganisms and pathogens [73,74]. Moreover, the action of SCFAs extends far beyond the gut, exerting pleiotropic functions in the entire organism and influencing several host processes, with a remarkable role in the host metabolism and epigenomic modifications [75]. Besides, SCFAs are shown to be a central mechanism of gut-brain communication. SCFAs may interact with the enteroendocrine cells and promote indirect signaling to the brain by the systemic circulation or vagal pathways, inducing the production of several hormones and neurotransmitters like GABA and serotonin in the gut. SCFAs are also related to a systemic regulation of inflammation and cytokine profile, being able to cross the BBB positively influencing its integrity and activating several mechanisms in the brain, modulating the levels of neurotrophic factors, neurotransmitters, neurogenesis, and reducing the neuroinflammation and glial dysfunction [76]. Thus, SCFAs may mediate a set of mechanisms potentially involved in the pathobiology of MDD.

Jiang et al. [77] compared the fecal microbiota composition in patients with MDD and healthy individuals. Despite a wide variety of phyla and specific bacteria being significantly altered in depressed versus non-depressed subjects, reduced levels of Faecalibacterium, an important butyrate producer was observed in patients with MDD, with a negative correlation with depression severity. In this line, Valles-Colomer et al. [78] also found that butyrate-producing *Coprococcus* spp. were also depleted in depression, being both Faecalibacterium and Coprococcus abundance directly associated with a higher quality of life indicators. Likewise, Wu et al. [79] compared the gut microbiome of depressed and non-depressed mice to explore the associations between these differences with SCFAs and neurotransmitters changes. They found that 29 different bacterial taxa can be found between depressed and non-depressed animals, remarking the abundance of the Allobaculum genus and Ruminococcaceae family. Significant differences in acetate, propionate norepinephrine and serotonin were reported, with a positive association between Allobaculum, acetate, and serotonin levels. Many studies have described the central action of SCFAs in serotonin production mainly due to their interaction with enteroendocrine cells [80]. In this sense, different mechanisms and specific microbial populations have been demonstrated. For instance, Engevik et al. [81] have recently studied the relevance of *Bifidobacterium dentium* and acetate in the release of serotonin both in rodent and human enteroids, which is also associated with an enhanced expression of the serotonin receptor 2a in the hippocampus in comparison to non-treated mice. Besides, spore-forming bacteria also appear to be critical modulators of serotonin production by enteroendocrine cells, prominently due to their effects in SCFAs production [82]. Biochemically, SCFAs appear to induce tryptophan hydroxylase 1 (tph1) in vitro, a rate-limiting enzyme implicated in serotonin production and the marker of neuroendocrine secretion chromogranin A by enteroendocrine cells [83].

A significant reduction of fecal SCFAs between depressed and non-depressed women was also proposed to be a potential contributor of suffering from MDD with a possible inverse association with depression severity [84]. Fecal measures of SCFAs also found a significant association between acetate, propionate, and butyrate levels with both depressive and gastrointestinal symptoms in young adults, therefore supporting that SCFAs are importantly related to the development of MDD [85]. SCFAs and particularly propionate production are associated with a decreased anticipatory response of the reward system, related to the monoamine dopamine [86].

As previously reviewed, altered dopaminergic neurotransmission is a major feature underpinning MDD, which is prominently related to the clinical manifestation of anhedonia one of the two main criteria of MDD [87]. Prior studies have proven the antidepressant effect of butyrate by reversing behavioral alterations in mouse models like anhedonia [88], low energy, cognitive and social abilities [76]. Similarly, the use of rifaximin was related to a decreased depressive-like behavior due to its favorable effects on Ruminococcaceae and Lachnospiraceae, positively correlated with butyrate production [89].

Two central mechanisms should be highlighted to understand the anti-depressant effect of SCFAs: (1) epigenetic regulation and (2) immunomodulatory action. Depressed patients show a prominent epigenetic footprint in comparison to non-depressed subjects, including critical alterations in DNA methylation, histone modifications, and non-coding RNAs (i.e., microRNAs) [90,91,92]. SCFAs act as histone deacetylases (HDAC) inhibitors, playing a key role in the DNA methylation, histone modification, and chromatin restructuring to alter gene expression [93]. For instance, SCFA-mediated inhibition of HDAC may lead to the hyperacetylation of histones H3/H4 and hence increase BDNF expression, showing antidepressant effects in mice [94]. This epigenetic modulation is mostly responsible for the immunomodulatory action of SCFAs. In the CNS, SCFAs are major mediators of microglial maturation and function whereas, in the gut, SCFAs induce the differentiation of Treg cells [93].

Overall, the epigenetic and immunomodulatory effects of acetate, and more prominently propionate and butyrate exert promising antidepressant effects, as they are mostly correlated with favorable microbial populations. Conversely, another type of SCFA, isovaleric acid has been shown to be correlated with certain bacterial populations related to MDD and augmented cortisol levels. A proposed mechanism of this pro-depressive effect of isovaleric acid is through interfering with synaptic neurotransmitter release [95]. Future studies should be destinated to deepen the different mechanisms of SCFAs in the pathology of MDD with a possible focus on some unexplored mechanisms like microRNAs.

#### 3.2.2. Lactate

Lactate is a crucial metabolic product of the anaerobic glycolysis involved in multiple physiologic and pathologic conditions due to its role as a relevant signaling molecule [96]. Despite not being considered as an SCFA, lactate is an important metabolite produced by some microorganisms including lactic acid bacteria, bifidobacteria, or proteobacteria and frequently it is converted into SCFA by several types of microorganisms [97]. Because of that, lactate is not abundantly found in the colon; however, it has been demonstrated that under physiological conditions, this metabolite can cross the BBB to match the energetic needs of the brain, influencing many neuronal functions such as excitability, plasticity, and memory consolidation [98]. The gut microbiota is not the only source of lactate, as this is also produced by the astrocytes in the brain, which is a local reservoir of lactate that transfers this metabolite to neurons and more prominently, by muscle cells during exercise [99].

The role of lactate in different brain disorders is undoubtedly complex. Bradley et al. [100] found that adolescents with MDD showed increased ventricular lactate, suggesting that mitochondrial dysfunction may be responsible for the augmentation of this component. Patients with bipolar depression (BD) also show an increased brain lactate detection in the brain that is ameliorated after treatment [101]. On the other hand, patients with severe depression show a significant detection of lactate in the urine in comparison to moderate and non-depressed subjects [102], therefore supporting the possible role of lactate in the etiopathogenesis of MDD. Conversely, lactate may also exert potential antidepressant effects in the brain by several mechanisms. One of them is related to the ability of lactate to revert the effects of stress on inducing depressive-like behavior, mainly supporting the hippocampal neurogenesis [103]. Lactate also promoted resilience to stress and rescued social avoidance and anxiety by restoring hippocampal HDAC levels and activity [104]. Similarly, the peripheral administration of lactate exerts antidepressant effects that were correlated with raised hippocampal lactate levels and changes in serotonin receptor trafficking, astrocyte functions, neurogenesis, nitric oxide synthesis, and cAMP signaling [105].

Overall, the role of lactate in the etiopathogenesis of MDD is poorly understood. However, a plausible explanation based on the available literature is that an accumulation of lactate in the brain due to an impaired mitochondrial function -a major feature of different psychiatric diseases- [106] or due to unknown mechanisms may be a cause or a consequence of MDD. Oppositely, increased levels of lactate in some critical brain regions like the hippocampus, possibly obtained from systemic sources like the microbiota or the muscles may be related to antidepressant effects. Physical exercise may be a powerful link between lactate and antidepressant effects influencing not only the muscle production of lactate but also the MGB axis [107].

#### 3.2.3. Tryptophan Metabolites

Tryptophan (Trp) is an essential and the least abundant amino acid with many bioactive effects in the body, especially through its multiple metabolites [108]. Gut microbiota is a key mediator of the actions of Trp. Despite some microbial species like *Escherichia coli* can synthesize Trp, there is not enough evidence supporting the relevance of gut microbiota. Conversely, the gut microbiota is critical for Trp metabolism, determining the benefits or harms [109]. The gut microbiota can convert Trp into a large list of products including small molecules like the antifungal pyrrolnitrin to long peptides with antibacterial activity as cyclomarin [110]. However, the most relevant metabolites derived from Trp with important actions for the MGB axis are (a) The transformation of Trp into the neurotransmitter serotonin, with benefits in the brain and gut function, and (b) the metabolism of Trp into kynurenine, tryptamine, and indole with neuroendocrine and immune-modulatory actions [111]. 5 bacterial phyla have been related to Trp metabolism, including Firmicutes, Bacteroidetes, Actinobacteria, Proteobacteria, and Fusobacteria. Of them, Clostridium, Burkholderia, Streptomyces, Pseudomonas, and Bacillus appear to be the most relevant genera involved in these events, according to an in silico study [112].

Because of the direct influence of Trp in serotonin synthesis, this amino acid may be pivotal for mood and cognitive modulation, hence supporting a possible role of Trp and its metabolism in the onset of MDD [113]. In this context, some studies demonstrate the benefits of certain types of bacteria involved in Trp metabolism. Tian et al. [114] reported how the presence of Bifidobacterium longum subsp. infantis E41 and Bifidobacterium breve M2CF22M7 appears to be related to an augmented level of 5-hydroxytryptophan, tph1, serotonin, and BDNF in the brain, significatively reducing depressive behavior. However, despite these effects, scientific evidence has failed to find promising and global benefits from increasing dietary Trp or by supplementation, which may benefit only some specific groups of patients who have a personal or family history of depression [115]. A potential explanation of this fact is that the gut dysbiosis found in patients with MDD may interfere with Trp metabolism and serotoninergic activity, as has been shown in some animal models [116]. In this line, it is widely accepted that an imbalance between the kynurenine pathway mediated by the enzyme indoleamine 2,3-dioxygenase (IDO) and serotonin synthesis may be a critical event of MDD. The pro-inflammatory environment in the gut leads to an increase in the activity of IDO, leading to enhanced production of some neurotoxic metabolites like 3-hydroxykynurenine and quinolinic acid and a decrease in serotonin synthesis [117]. Likewise, it seems that enhanced IDO activity may be a prominent pathophysiological mechanism related to the inflammation-induced depressive-like behavior by endotoxemia [118].

Interestingly, these alterations may also be responsible for the hyperactivation of the HPA axis [117]. These results are in consonance with some systematic reviews and meta-analysis that showed that patients with MDD displayed reduced levels of Trp, kynurenine, and kynurenic acid -two components of this pathway with neuroprotective effects-; low kynurenine to Trp ratio-supporting the imbalance between kynurenine route and serotonin synthesis- and an augmented detection of different neurotoxic substances from this pathway like quinolinic acid [119,120]. Therefore, dysregulation of the Kynurenine pathway with an increased neurotoxic/neuroprotective ratio appears to be a critical pathophysiological mechanism involved in MDD.

Apart from serotonin depletion, the aberrant kynurenine pathway is also related to abnormal glutamatergic neurotransmission [121] and more worryingly, with neuroinflammation. Tryptophan, kynurenine, 3-hydroxykynurenine, and cytokines may cross the BBB. In the brain, cytokines, and glucocorticoids enhance the kynurenine pathway, driving to an increased formation of the neurotoxic quinolinic acid in the microglia and infiltrated macrophages, favoring the neuroinflammatory response [122]. Many of the kynurenine metabolites exert their immunomodulatory action through the activation of aryl hydrocarbon receptors (AHR) and that single nucleotide polymorphisms (SNPs) affecting this gene may be involved in the development of different immune and inflammatory diseases [123]. In patients with MDD, SNPs affecting AHRs were associated with interindividual variations in the plasma levels of kynurenine that in turn was associated with MDD severity [124].

Moreover, the kynurenine pathway may also interfere with the tryptamine activity in the brain [121]. The function of tryptamine in the brain is not fully understood yet, although it is hypothesized that it may play a possible neurotransmitter or neuromodulatory role [125]. In the gut, different microorganisms are able to synthesize tryptamine through the enzyme tryptophan decarboxylase. An increased tryptamine production may be related to reduced Trp bioavailability and serotonin synthesis in the brain [126]. Finally, Trp may also be converted into indole, indican, and skatole as well as indole acid derivatives by the gut microbiota. These metabolites as well as tryptamine are also important ligands of AhRs [127]. Jaglin et al. [128] demonstrated that chronic overproduction of indole by gut microbiota was related to anxiety- and depressive-like behaviors in rats, possibly by augmenting brain levels of oxindole and through the activation of the vagus nerve. Conversely, it seems that certain types of indoles produced by the gut microbiota may exert neuroprotective effects like indole propionic acid [129]. Because of that, the analysis of indole and indolic derivates as blood biomarkers in different disorders like MDD is receiving growing attention [130].

Overall, compelling evidence is supporting the relevance of gut microbiota-derived Trp metabolites in the etiopathogenesis of MDD. However, further efforts to unravel the role of Trp metabolites in depression are still needed, especially regarding tryptamine, indole, and their derivates.

#### 3.2.4. Microbial Influence in Other Amino Acids

Not only Trp but also other amino acid metabolism appears to be tightly linked to the gut microbiota, with noteworthy implications for patients with MDD. Glutamate and phenylalanine are some amino acids significantly altered in body fluids of depressed subjects [131,132,133]. Gut microbiota plays a pivotal role in the regulation of both amino acids. Regarding glutamate, many bacteria consume this amino acid to generate energy or to form their structural components. Simultaneously, other bacteria can produce glutamate and some species may synthesize GABA from glutamate [134]. Among the main glutamate producers, it should be highlighted the role of coryneform and lactic acid bacteria whereas some gram-positive and gram-negativee bacteria expressions transform glutamate into GABA through the enzyme glutamate decarboxylase (GAD) [135]. Lactobacillus and Bifidobacterium are two central genera involved in the production of glutamate and GABA. Altaib et al. [136] found an interesting association between the fecal abundance of the Bifidobacteriaceae family and GABA levels in healthy individuals. *Bifidobacterium dentium* appears to be a pivotal microorganism involved in GABA production from glutamate, glutamine, and succinate, which was also related to an augmented tyrosine production [137]. *Bifidobacterium adolescentis*, and particularly the strains PRL2019 and HD17T2H also seem to be major producers of GABA in the gut [138] whereas Bravo et al. [139] studied that the administration of Lactobacillus rhamnosus (JB-1) induced through vagal pathway noteworthy alterations of GABA receptor expression in different brain regions related to MDD, including cortical structures like the cingulate and prelimbic regions, the hippocampus, amygdala, and locus coeruleus. Besides, Bacteroides Parabacteroides and Escherichia species are also importantly involved in the synthesis of GABA, with a negative correlation between fecal Bacteroides and brain signatures associated with depression [140]. In this line, a recent study conducted by Otaru et al. [141] has shown *that Bacteroides* spp. is a critical agent involved in the GABA production due to the activation of the GAD enzyme in a pH-dependent manner, acting as a protective mechanism against acid stress in these microorganisms. Collectively, gut microorganisms are key mediators of glutamate and GABA production, with important neurotransmitter activity in the brain. Similarly, phenylalanine, another product also synthesized by some microorganisms, is the precursor of the catecholamines epinephrine, norepinephrine, and dopamine, which are involved in the pathogenesis of MDD and therapy resistance [142].

Studies of the fecal metabolome and microbiota in chronic unpredictable mild stress (CUMS) rats, an animal model of depression reported consistent changes in the levels of many amino acids like threonine, isoleucine, alanine, serine, tyrosine, and oxidized proline, thereby suggesting that alterations in the gut microbiota are linked to changes in circulating amino acids and depressive behavior [143]. In this line, Kawase et al. [144] compared the serum levels of different amino acids in specific pathogen-free (SPF) and germ-free (GF) mice. Interestingly, they claimed that the concentration of different amino acids in the host brain seems to be dependent on the presence of the gut microbiota, concluding that amino acid metabolism in the host brain could be modified only by manipulating gut microbial communities. Moreover, Steffens et al. [145] reduced levels of phenylalanine, aspartate, glutamate, and serine were found in depressed individuals with heart failure in comparison to patients without MDD, although the role of heart failure in these differences was unclear.

Collectively, almost all the amino acid levels were altered in animal models and human patients of MDD [134]. Gut microbiota is likely a central mediator of these changes, participating in the synthesis and metabolism of many amino acids. Future studies are warranted to understand the precise actions of gut microbiota in these components and how it could be managed.

#### 3.2.5. Secondary Bile Acids

Bile acids (BA) are crucial physiological agents with prominent functions in digestion, nutrient absorption, and the elimination of lipids, toxic metabolites, and xenobiotics. BA are key regulators of the gut microbiota composition and in turn, gut microbiota metabolizes BA, determining the bile acid pool size [146]. Primary BA is produced by the liver and secreted into the small intestine whereas secondary BA corresponds to the metabolism of these products by the gut microbiota. Cholic acid (CA) and chenodeoxycholic acid (CDCA) are the two major primary BA and they are frequently conjugated with taurine or glycine for secretion into bile [147].

On the other hand, secondary BA is formed by different reactions including the biotransformation and hydrolysis of conjugated BA to free BA and taurine or glycine thanks to the action of the bile salt hydrolase (BSH); 7α-dehydroxylation of the CA or CDCA leading to the conversion of deoxycholic acid (DCA) and lithocholic acid (LCA), the epimerization of CDCA into ursodeoxycholic acid (UDCA) and subsequently the 7β-dehydroxylation of UDCA yielding LCA [148]. Most BSH bacteria are Gram-positive gut bacteria including *Clostridium*, *Enterococcus*, *Bifidobacterium*, and *Lactobacillus*. The genus Bacteroides is the only Gram-negative bacteria with BSH, and some archaea like *Methanobrevibacter smithii* and *Methanosphera stadmanae* may also have BSH activity [149].

The dehydroxylation of BA is also conducted by many bacteria from the Bacteroides and Clostridia genus, and in *E. coli*, *C. testosteroni*, and *Ruminococcus* spp. [150]. Approximately, 95% of the BA is reabsorbed in the terminal ileum and returned to the liver via enterohepatic circulation, including secondary BA [151]. However, BA also exerts multiple metabolic functions in the body as well as acting as signaling molecules due to their binding to two main receptors: Farnesoid-X-Receptor (FXR) and the G-Protein-Coupled Bile Acid receptor-1 (GPBAR-1, also known as TGR5) [152]. Therefore, secondary BA influences a plethora of modulatory functions not only at local but also at systemic levels.

In this sense, previous studies have supported that secondary BA is a critical molecule involved in the MGB axis. The brain may also present its BA pool, and despite the brain may synthesizing its BA, it is mostly suspected that BA acts as a communication bridge between the gut microbiota and the brain [153]. This statement is supported by growing evidence that describes a worrying association between an aberrant BA communication and the development of neurodegenerative diseases [154,155,156]. There are two main pathways by which BA may influence the brain: (1) BA may cross the BBB and bind to FXR and TGR5 receptors in the brain and (2) Activation of the intestinal FXR and TGR5 receptors can result in the release of fibroblast growth factor 19 (FGF19) and glucagon-like peptide 1 (GLP-1), leading to indirect signaling of BA to the brain [157].

Physiologically, the effects of BA in the brain are mainly mediated through GLP-1. However, under pathological conditions, the activation of the FXR may play a central role in the onset of different psychiatric disorders like MDD. In this sense, an overexpression of hippocampal FXR seems to cause depression-like symptoms and reduced BDNF levels in rats [158]. Conversely, the activation of TGR5 by the secondary bile acid tauroursodeoxycholic acid (TUDCA) and other receptors in the brain of BA like vitamin D receptor (VDR) and pregnane X receptor (PXR) activated by LCA appears to present antidepressant effects, thereby suggesting that the prodepressant or antidepressant effects of secondary BA in the brain may be dependent of the receptor in which they act, although this hypothesis must be confirmed [159].

Nowadays, the action of secondary BA on the pathogenesis of MDD remains to be fully elucidated. Simultaneously, the use of some antidepressant drugs such as paroxetine -a selective serotonin reuptake inhibitor (SSRI)- seems to alter gut microbiota and BA production in mice and it is unknown if these changes may be counterproductive for patients with MDD [160]. Further efforts are needed to unravel the relevance of secondary BA in MDD.

#### 3.2.6. Vitamins

Vitamin deficiencies are frequently observed in patients with MDD, especially vitamin D and those from the B complex [161,162]. Vitamin D regulates gut microbiota composition through several mechanisms whereas gut microbiota may synthesize a wide variety of B vitamins and vitamin K [163,164]. Thus, despite vitamin D supplementation being an interesting approach to improve gut microbiota in patients with MDD, in this section we will explore the importance of B vitamins as a metabolite produced by intestinal bacteria.

Low levels of vitamin B1 (thiamine), B2 (riboflavin) B3 (niacin), B6 (pyridoxine) B7/8 (biotin), B9 (folate) and B12 (cyanocobalamin) are pivotal B vitamins associated with MDD [165,166]. However, despite B vitamin supplementation having provided some benefits for healthy individuals and at-risk populations for stress, the evidence has failed to support the use of these vitamins for ameliorating depressive symptoms [167]. A potential explanation may rely on the fact that gut dysbiosis impairs the synthesis of these vitamins [168]. Thus, the metabolic production of B vitamins from the gut microbiota is crucial for the proper functioning of the brain. Indeed, several studies are confirming the benefits of using certain vitamins in combination with probiotics, frequently represented by different species and strains of Bifidobacterium and Lactobacillus [166]. For instance, Reininghaus et al. [169] proved the effects of combining probiotic plus biotin supplementation for four weeks in patients with MDD. They reported that in comparison to the groups that only received vitamin B7 plus placebo or probiotics plus placebo, patients supplemented with both probiotics and vitamin showed an increased synthesis of vitamin B6 and B7 and improved clinical outcomes. Thus, the gut microbiota is crucial for determining the body levels of B vitamins, but an adequate dietary context is needed to obtain the benefits from those bacteria involved in vitamin synthesis, with noteworthy positive outcomes for the brain.

#### 3.2.7. Choline-Derived Metabolites

Choline metabolites are an example of the importance of gut microbiota and their metabolites in mental health. Choline is a nutrient especially found in meat, milk, grain, eggs, and their derived products and fish [170]. Choline may be transformed into a wide variety of relevant metabolites like trimethylamine (TMA), betaine, phosphocholine, and acetylcholine. The gut microbiota plays a critical role in choline metabolism, is importantly involved in TMA production and then it is transformed into trimethylamine-N-oxide (TMAO) in the liver [171]. Moreover, there are some bacteria capable of using betaine obtained from dietary sources (i.e., dark green leafy vegetables, whole grains, or seafood) like *Akkermansia muciniphila* leading to some favorable effects due to the increase of SCFAs production and restoring gut microbial populations [172,173]. Both choline and betaine have been recognized as central biomarkers of cognitive performance [174,175]. Likewise, altered levels of choline in different brain regions have been associated with MDD [176,177], and betaine has been identified as a novel adjunctive therapy for improving the antidepressant effects of ketamine while preventing some its main adverse effects [178]. However, there is some evidence supporting that the gut microbiota status and the TMA/TMAO levels rather than choline or betaine may be better indicators of the favorable or detrimental effects of these metabolites [179]. Main bacteria involved in TMA production are included in the Firmicutes phyla, especially the Clostridium cluster XIVa and Eubacterium strains, but also some Actinobacteria and Proteobacteria [180]. Gut dysbiosis is harmfully associated with increased TMA and TMAO production, which may be accumulated in the different tissues and affect different organs and systems in the body, particularly the cardiovascular system and the liver [181]. Indeed, previous studies have found a positive relationship between TMAO levels and the severity of depressive symptoms in females and males [182]. In addition, Romano et al. [183] denoted that raised levels of choline-consuming bacteria may have detrimental effects on the global DNA methylation patterns in mice, as choline betaine are involved in regulating the host metabolism epigenetics and behavior.

All in all, future studies are needed to unravel the role of the gut microbiota in the mediation of choline, betaine, and TMA/TMAO effects in the brain of patients with MDD.

#### 3.2.8. Estrobolome

The last point explored in this section is a novel but interesting approach that may serve to understand the pathogenesis of MDD in women: The estrobolome. This term refers to a set of bacteria that are able of metabolizing and modulate the estrogen levels in the body. Estrogen is transported in the blood through the sex hormone-binding globulin (SHBG), which is produced by the liver and determines the proportion of estrogen-free (Biologically active) or bound (Inactive) [184]. Free estrogen activates two distinct nuclear receptors, estrogen receptor alpha (ERα) and estrogen receptor beta (ERβ), virtually regulating all biological processes [185].

The gut microbiota is a major determinant of the circulating levels of estrogens through different mechanisms. Firstly, they produce and secrete the enzyme β-glucuronidase, which deconjugates estrogen and increases the bioavailability of its active form [186]. Furthermore, some microorganisms break down indigestible dietary polyphenols to synthesize estrogen-like compounds or estrogen mimics that exhibit varied estrogenic potency [187]. In men and postmenopausal women, gut microbiota diversity and functions influence the levels of non-ovarian estrogens via enterohepatic circulation, where Clostridia taxa and genera from the *Ruminococcaceae* family could be key microbial populations involved in this regulation [188]. Likewise, estrogen levels influence the growth of certain types of bacteria as well as the consumption of the above-mentioned polyphenols, thus indicating the bidirectional role of the gut microbiota and estrogens [189]. On the other hand, gut dysbiosis may significantly impair the action of gut microbiota in the estrogen metabolism, driving the reduction of circulating estrogens, which is associated with a plethora of adverse outcomes ranging from obesity, metabolic syndrome, malignancies, endometrial hyperplasia, endometriosis, polycystic ovary syndrome, fertility, cardiovascular disease, and cognitive dysfunction [190].

The evidence supporting the role of altered sexual hormones in the pathogenesis of MDD in women could be found in the existence of three different types of depression related to the menstrual cycle: Premenstrual dysphoric disorder (PMDD), postpartum depression, and perimenopausal depression. The use of estradiol, but not progesterone appears to be effective for PMDD, postpartum, and moderate/severe perimenopausal depression [191,192]. These benefits may arise from the neuroprotective effects of estrogens mediating some neuroplastic actions in the hippocampus and regulating serotonin receptors levels. However, the use of estradiol as the first-line treatment is not supported by current scientific evidence [193]. Instead, targeting the estrobolome in the prevention and treatment of these specific disorders could be an effective interesting approach, as estrogen levels may be directly influenced by the gut microbiota and women may benefit from the actions of these bacteria. It is also of note that some studies have reported that lower levels of testosterone but not estrogen were associated with more severe depressive symptoms in a group of 23 women with MDD [194]. In this context, Markle et al. [195] reported that gut microbiota transplantation from male to female mice led to an augmentation of testosterone levels and metabolomic changes in females and future studies may consider the benefits from using testosterone-producer microorganisms to raise testosterone levels.

Considering the current evidence, as gut microbial diversity is directly related to the levels of non-ovaric estrogen levels and the intake of certain dietary components like polyphenols may aid in the modulation of beneficial microbial communities and ER activation, nutritional and lifestyle interventions may be a potential strategy to consider for boosting the estrobolome. Some examples of this strategy will be later discussed in the following section.

## 4. Translational Applications for the Depressed Patient

### 4.1. The Importance of Boosting Individual’s Microbial Metabolome

It is undeniable that every person harbors a unique gut microbiota that results from their genetic factors and history of epigenetics throughout life, implying seasonal and temporal variations as well [196,197]. Moreover, the development of certain body systems, especially the brain, coincide with the periods of major change in the microbiota, denoting the importance of microbiota establishment in the influence on epigenetic modifications shaping CNS, which could be interesting as “windows of opportunity” for disease prevention [29,198].

For these reasons, each patient’s approach must be personalized. Each individual’s alterations in microbiome explain the different individual’s responses to pharmacologic agents, hence talking not only about pharmacogenetics and pharmacogenomics; but also about “*pharmacomicrobiomics*”, as the different drug action and effect due to microbiome variations [199,200]. To standardize human biome types is also not an easy task due to the wide variety of across the different geographical areas and the different socioeconomic standards now that even two people from the same place with the same job and lifestyle may have many differences in their microbiomes and both may be perfectly healthy [201].

In all this context, it has been crucial to rely on systems biology, especially to study the interactions of microbial and host metabolomics determining the physiological characterization in health and disease based on plasticity and diversity of microbes and specific metabolites [202,203]. The challenge here resides in understanding how microbially produced metabolites subjected to the influence of diet and drugs can modulate epigenetic mechanisms and link them to disease risk [204,205]. Despite individual variations in health status, certain related-pathology patterns are repeatedly observed, and thus, the “*Microbiome Wide Association Studies*” (MWAS), mapping the microbiome, are providing new biomarkers of disease. These analyses allow clinical medicine to keep on the way towards integrating traditional clinical epidemiology with microbial one [206]. Nevertheless, there is still so much effort to put into the data analysis at this level, and on introducing algorithms in health systems to assess patients this way.

Furthermore, although colon microbiota has some differences with fecal, the latter keeps being useful for sampling methods, being non-invasive, and seems helpful in the discovery of pathology biomarkers. New sampling technologies are trying to find non-invasive solutions to palliate these differences [207]. Lessons from animal models have allowed the study of metabolites produced by representative gut microbiota in MDD. To target gut dysbiosis in depressed patients, the strategy to follow can be stimulating the diminished beneficial bacteria metabolism over not desired ones, being both diagnosis and treatment biomarkers. For instance, we already mentioned that in fecal microbiota from patients with MDD, Enterobacteriaceae and *Alistipes* are found over-represented whereas butyrate producer *Faecalibacterium* is underrepresented and negatively correlated with the severity of depressive symptoms [77].

But to reconstruct the depressed patient’s eubiosis, integrative therapies are required. Preferred counseling for targeting gut microbiota are probiotics and prebiotics, and/or food containing these [11]. Today, there are also potential pharmaceutical products grouped under the name of “*pharmabiotics*”, that join several probiotics, prebiotics, and nutraceutical compounds in a single food supplement [208]. New advances into holistic treatments for mental disease are being estimated due to the multidimensional system in which the gut microbiome is involved (immune, endocrine, CNS, HPA, Trp metabolism, neurotransmission…) [209]. In the following sections, we discuss complementary therapies including not only oral adjuvant treatments but also recommendations in lifestyle factors like diet, exercise, or sleep, that profoundly shape microbiota mechanisms and hence, host epigenetics pathways.

### 4.2. Emulation of Microbial Metabolism

#### 4.2.1. Adjuvant Therapy

##### Supplementation with Probiotics

Moreover, evidence reports probiotics have denoted effectiveness in reducing depressive symptoms when combined with antidepressants and ameliorating the coexisting metabolic disorders, but there are no standardized recommendations for MDD [210]. Popularly known, *Lactobacillus* and *Bifidobacterium* have been the most extensively used genera for the restraint of undesired bacteria (i.e., overgrowth of *Escherichia coli* or the opportunistic infection of *Citrobacter rodentium*) and the strengthening of intestinal epithelia in gastrointestinal disorders. But more specifically, Skonieczna-żydecka et al. [211] reviewed beneficial bacteria “psychobiotics” that had reunited documented efficacy for the treatment of MGB and HPA axis disorders, concretely for neutralizing anxiety and depressive symptoms: *Lactobacillus fermentum NS8 and NS9*, *Lactobacillus casei Shirota*, *Lactobacillus gasseri OLL2809*, *Lactobacillus rhamnosus JB-1*, *Lactobacillus helveticus Rosell-52*, *Lactobacillus acidophilus W37*, *Lactobacillus brevis W63*, *Lactococcus lactis W19 and W58*, *Bifidobacterium longum Rosell-175*, *Bifidobacterium longum NCC3001*, *Bifidobacterium longum 1714*, *Bifidobacterium bifidum W23*, *Bifidobacterium lactis W52*, and *Lactobacillus plantarum 299v*.

These neurobiotics entail bacteria that exert neuroactive metabolite synthesis and modulate neurotrophic factors against MGB axis stress [211]. These would be SCFAs-producing bacteria and neuroactive compounds producing bacteria. Lessons from animal models show the biotechnological advances in genetic engineering of these new strains. The example of *Bifidobacterium breve CCFM1025* resulted in increased expression of BDNF, restoration of chronic stress-induced gut microbiota, and the increase in SCFAs and serotonin production [212]. Chronic treatment with new strains of *Lactobacillus plantarum*, like *Lp 286*, also exposed anti-depressant- and anti-anxiolytic-like effects in mice but needed further investigation about GABA mediation [213].

Commercial formulas, i.e., Ecologic^®^ barrier (*B. bifidum* W23, *B. lactis* W51, *B. lactis* W52, *L. acidophilus* W37, *L. brevis* W63, *L. casei* W56, *L. salivarius* W24, *Lc. lactis* W19, *Lc. lactis* W58) (NCT04951687) including multispecies have been proved on memory performance and cognitive reactivity to sad mood using the Leiden Index of depression sensitivity scale, tested in randomized human trials, denoting significant reduced depressive-like behavior in mild to moderately depressed patients.

More evidence from clinical trials with the multispecies combination is needed to standardize methods for clinical practice guidelines of MDD. One controlled trial with multispecies supplementation (*Bifidobacterium bifidum W23*, *Bifidobacterium lactis W52*, *Lactobacillus acidophilus W37*, *Lactobacillus brevis W63*, *Lactobacillus casei W56*, *Lactobacillus salivarius W24*, *and Lactococcus lactis* (*W19 and W58*)) focused on prevention, administered the formula to non-depressed individuals; then, evaluating their behavior, 4 weeks after the intervention they showed significantly reduced rumination, aggressive thoughts and balanced cognitive reactivity to a sad mood [214]. These effects may be due to their association with OXT and AVP, two critical neuropeptides involved in the pathogenesis of MDD. Previous studies have described that gut microbiota may regulate OXT levels in the brain, and as OXT may bind to the AVP receptor, the gut microbiota can influence the actions of both neuropeptides [215]. *Lactobacillus reuteri* has been shown to be one of the most important regulators of OXT levels in the hypothalamus, with favorable effects on social behaviors [216]. Besides, a recent study shows that this effect was accomplished even by using a non-viable lysate of this microorganism [217]. Thus, it is possible that some undiscovered peptides or metabolites produced by *Lactobacillus reuteri* and other bacteria may directly regulate the levels and actions of OXT and AVP.

In *Clinical Trials* website we can already find the trials that are being accomplished for MDD treatment, some of them combined with prebiotics and some with common antidepressants: *Ganeden BC30* (*Bacillus coagulans GBI-30*) (NCT01337609); multi-strain probiotic *BioKult* (*Bacillus subtilis*, *Bifidobacterium bifidum*, *Bifidobacterium breve*, *Bifidobacterium infantis*, *Bifidobacterium longum*, *Lactobacillus acidophilus*, *Lactobacillus delbrueckii* ssp. *bulgaricus*, *Lactobacillus casei*, *Lactobacillus plantarum*, *Lactobacillus rhamnosus*, *Lactobacillus helveticus*, *Lactobacillus salivarius*, *Lactococcus lactis* ssp. *lactis*, *Streptococcus thermophilus*) (NCT03893162); *Lactobacillus Plantarum PS128* (NCT03237078); *Probio’Stick* (*Lactobacillus helveticus + Bifidobacterium longum*) (NCT02838043); *Lactobacillus Plantarum 299v* + sertraline + escitalopram (NCT02469545) among others. Their objective is also testing symptoms besides metabolites (tryptophan, kynurenic pathway), cortisol concentration, and pro- and anti-inflammatory cytokines.

In depressed patients, there are repeatedly observed shifts in the relative abundance of more bacterial genera like *Akkermansia*, *Ruminococcus*, or *Prevotella* spp. These appear decreased and correlate with behavioral metrics of depression and anxiety [218]. Also, the under-representation of *Faecalibacterium* and over-representation of *Actinobacteria* and *Enterobacteriaceae*, are related to clinical characteristics and metabolic markers in MDD and bipolar disorder [219]. Specific strains like *Akkermansia muciniphila* and *Faecalibacterium prausnitzii*, have been studied in vitro with human epithelial colorectal cells, showing an impact on the expression of serotonin system-related genes [220] like the up-regulation of *SERT* gene (serotonin transporters) [221]. So, these two are promising probiotics also as potential modulators of the homeostasis at the serotonin system level.

Interestingly to mention is that certain antidepressant drugs like fluoxetine, escitalopram, venlafaxine, duloxetine, or desipramine can alter relative abundances of different strains in gut bacteria, so the approach here is to optimize these antidepressants to not affect the microbial composition or the suggestion of supplementation with those bacteria that become diminished by the drug. For example, *Ruminococcus flavefaciens* in preclinical models as a potential probiotic seems to depressive-like behavior by ameliorating mitochondrial oxidative phosphorylation in brain networks, boosting antidepressant efficiency as well [222].

Psychobiotics are the new field to explore with large marketing behind. Safety and efficacy studies are still needed, just as probiotics’ effectiveness has not been widely supported as they do not have the immediate effects medicines may offer [223]. However, Yong et al. [224] allege that the advantages presented for probiotics over standard antidepressants include the absence of secondary effects and the stigma involved Many effects of probiotic strains are also summarized in their review [224]. Apparently, in the era of precision medicine in which microbiota-directed therapy has been included, more new terms related to probiotics are arising, like “postbiotics”, alluding to the molecular signature of probiotics [225]. Furthermore, “pharmabiotics” consist of a new concept of biologically active therapeutics, comprising dead organisms, their components, and bioproducts [208].

Although this line of research is not directly included as “probiotics”, the basis of fecal microbiota transplantation (FMT) is quite similar. Simply, FMT consists of transferring stool from a healthy donor into the colon of a patient with an established pathology related to an altered microbiota with the aim to restore the normal microbiota and cure or ameliorate the disease [226]. In general views, FMT could be considered a safe therapeutic method with few adverse effects, although there are two main limitations to address before being recommended for patients with MDD. The first is related to the fact that there is still some limited knowledge about the long-term outcomes of FMT. It is still needed great efforts to personalize FMT for different patients and conditions according to varied hosts and diseases [227]. The second issue is related to the donor. Compelling evidence is supporting that FMT has the potential not only for ameliorating depressive or anxiety-like symptoms when healthy gut microbiota is transferred but also for inducing or aggravating the symptoms if the donor shows an aberrant gut microbiota and depressive behavior [228]. It is essential to fulfilling a thorough examination of the donor with a questionnaire, interview, blood tests, and stool analysis before allowing the transplantation [227]. However, because every individual exerts a unique gut microbiota footprint and the current limitations above described, we think that there is still a long road to cover before considering the application of FMT for the treatment of MDD.

##### Supplementation with Prebiotics

Following the International Scientific Association for Probiotics and Prebiotics (ISAPP), a prebiotic is a substrate that is selectively utilized by host microorganisms, that stimulate the growth and/or activity of these in the digestive system, while conferring a health benefit and reducing the risk of diseases mediated by microbiota aberrations [229]. In general, prebiotics is non-digestible carbohydrates (fructans, galactooligosaccharides, starch, and others), although the current definition considers the use of other non-carbohydrates compounds as prebiotics [230]. Some of the mechanisms proposed of the prebiotics in the microorganisms are that they may aid in the production of energy, metabolites, and micronutrients in the microorganism which may be relevant for the host, also allowing the growth of certain groups of beneficial bacteria [230,231].

A recent systematic review and meta-analysis showed that contrary to probiotics, prebiotics alone did not exert any significant differences in depressive or anxiety symptoms when compared to placebo [232]. However, this fact does not implicate prebiotics are completely unusefulness for MDD. Prebiotics are bioactive compounds that ease helpful bacteria in the gut and from probiotics to bloom. They might be a good option to fortify formulas with certain psychobiotic/probiotic strains. The term “symbiotic” refers to that combination of pre and probiotic. Noonan et al. reviewed patients with co-morbidities like Intestinal Bowel Syndrome can get better benefits from pre/probiotic treatments but that there is still a lack of studies with larger sample sizes and duration to support their effects on anxiety and depression [233]. Some of the proposed mechanisms of the antidepressant effects of prebiotics are the following: (1) Enhanced SCFAs and Treg in the gut; (2) Decreased plasma corticosterone and (3) Augmented activity of the prefrontal cortex, BDNF, and serotonin accumulation while decreasing amygdala activity, reduced IDO expression, cytokines detection in the brain and microglial activation [234]. However, there is still little evidence supporting the real benefits of these actions, and as has been above-mentioned, to achieve a significant antidepressant effect, prebiotics must be combined with probiotics.

Nowadays, prebiotics/probiotics and symbiotics are starting to be employed, not only in form of a specific formula but also in foods, beverages, and even in topical products like toilet paper [235,236]. Following the scientific evidence here, it should be importantly noticed that a food or beverage does not present any benefit itself by presenting any prebiotic/probiotic/symbiotic. Instead, the whole composition of the food/beverage, the interactions between those components (both facts can be grouped under the term “food matrix”) and the degree of processing are the most relevant factors to determine the healthy or unhealthy effects of the food [237]. In this sense, fermented foods may be one of the most interesting approaches from the use of prebiotics/probiotics/symbiotics in the food, although the results of the real efficacy of fermented foods/beverages are quite heterogeneous [238,239]. Preclinical studies show promising, but still inconsistent results of the antidepressant effects of fermented foods, due to their probiotic, prebiotic, and biogenic actions [240].

Future studies are warranted first to unravel the antidepressant mechanisms of prebiotics and symbiotics in patients with MDD, to find an adequate combination of both, and to explore the most appropriated vehicle (in formula or fortified non-ultra-processed foods and drinks).

##### Supplementation with SCFAs and Derivatives

SCFAs oral administration has proved evidence as a potential microbiota-targeted therapy for stress-related disorders. In a mice model, a mixture of acetate, propionate, and butyrate could promote anxiolytic and antidepressant effects, with ameliorated corticosterone levels, although changes in caecal microbiota composition were not registered, as well as colonic gene receptors for those fatty acids were not upregulated [88]. In a study conducted by Müller et al. [85], in fecal samples from depressive people, the correlation of shifts in SCFAs ratio with depressive symptoms was positive with acetate levels and negative with butyrate and propionate. SCFAs as nutrabiotics may therefore seem interesting for treatment.

Preclinical models have tested the effects of epigenetic drugs like sodium butyrate (NaBut), which is a histone deacetylase inhibitor and has chemical similarities with SCFA butyric acid. NaBut denoted antidepressant-like effects by promoting a decrease of methylation in BDNF, therefore supporting its upregulation, and then having effects in neurogenesis and neuroplasticity [241]. The observation is that NaBut can ameliorate chronic stress and cognitive related proteins at the hippocampus [242], improving memory function [243,244], and also has been observed increased activity in tricarboxylic acid cycle enzymes from MDD-associated mitochondrial dysfunction [245].

For its part, acetate supplementation in mice promotes antidepressant-like effects also at epigenetic levels by increasing histone acetylation with consequent down-regulation of BDNF, enhanced dendritic branches, and spine density of hippocampal pyramidal neurons, hence improving synaptic plasticity in the hippocampus [246]. Similarly, daily vinegar (acetic acid) ingestion was clinically tested in healthy adults, showing improvements in depression scores and amino acids metabolisms (increased metabolism of glycine, serine, threonine) [247].

Eventually, there is also evidence from preclinical studies with other derivative salts from propionate. In a mice model, sodium propionate effects are dose-dependent, being an antidepressant when administered at a low dose and pro-depressant when administered at a very high dose (reduction of norepinephrine, dopamine, and Trp; and increase of GABA, Kynurenine, and 3-hydroxykynurenine). Due to the observed disruption in the homeostasis of neurotransmitters, in the same study, Hao et al. warned about the potential danger of propionate as a commonly used food additive [248].

##### Supplementation with Nutraceuticals

Nutraceuticals are foods or part of them with multiple physiological actions in the body, aiding to prevent the onset of different pathologies and showing a promising role as adjunctive therapy in established diseases [249]. Scientific evidence gives a central role of vitamin D, S-adenosylmethionine (SAMe), omega 3 polyunsaturated fatty acid (ω-3 PUFA), and methylfolate is the most relevant antidepressant nutraceuticals [250]; although it is being currently explored the antidepressant actions of other nutraceuticals like creatine, amino acids, micronutrients, and some plant-derived bioactive compounds [251]. Most of these nutraceuticals have significant effects on the gut microbiota, and simultaneously, gut microbiota may be responsible for some of the benefits reported by nutraceuticals.

Vitamin D is an example of the synergic actions between gut microbiota and nutraceuticals. On the one hand, vitamin D has direct actions in the gut microbiota. Singh et al. [164] recently discovered that vitamin D supplementation leads to an increase in microbial diversity, with an increase of Bacteroidetes phyla, reduced Firmicutes/Bacteroidetes ratio, and enhancing some microbial populations with potential health benefits like *Akkermansia muciniphilla* and *Bifidobacterium* spp. among other changes. These changes in the microbiota composition may lead to an augmented production of some microbial metabolites like SCFAs which exert similar and synergic immunomodulatory actions like vitamin D, enhancing their benefits [252].

SAMe is a metabolic product synthesized during the cycle of methionine, is considered the universal methyl donor in living organisms [253]. In other words, SAMe has considered one of the major epigenetic regulators discovered through its actions in the one-carbon cycle [254] and the gut microbiota may also play a crucial role in these favorable actions. Jabs et al. [255] described that gut microbiota composition may affect the metabolism of SAMe. More detailed, *Akkermansia muciniphila* and *Lactobacillus plantarum* were able to lead epitranscriptomic modifications in the host, hence regulating the role of SAMe in many epigenetic reactions. In turn, mice fed with high levels of methionine, the precursor of SAMe lead to an impairment in gut microbiota composition, causing small bowel bacterial colonization and increased the abundance of pathogenic bacterial species Burkholderiales [256].

5-methyltetrahydrofolate (5-MTHF) is the active form of vitamin B9 (folate). This component is essential for restoring SAMe levels in the one-carbon cycle [257]. The gut microbiota is a pivotal source of folate production in the gut and some patients may benefit from the actions of microbial folate, as some patients fail to transform the folate into their activities. Particularly, those with single-nucleotide polymorphisms (SNPs) in dihydrofolate reductase (DHFR), which is required to convert folic acid to THF are not able to use folate supplements and target the gut microbiota (i.e., with probiotics) and their folate production could be essential to ensure the actions of 5-MTHF [258].

Omega 3 PUFA is a major structural and functional component of the brain, with many antidepressant mechanisms, described [251]. Among them, omega 3 PUFA exerts important prebiotic actions in the gut, is associated with a decrease in Faecalibacterium, an increase in the Bacteroidetes phyla, and butyrate-producing bacteria belonging to the Lachnospiraceae family, has been observed [259].

Among the most interesting plant-based compounds with antidepressant effects related to the gut microbiota, we must consider dietary polyphenols. Indeed, currently, the symbiotic use of probiotics with polyphenols is being widely explored in the management of MDD, as both combined seem to ameliorate neuroinflammation, microglial and inflammasome activation, reduce OS and balance serotonin metabolism [260]. In this sense, anthocyanins, a specific group of polyphenols participate in the regulation of the MGB axis by regulating host tryptophan metabolism, increasing the production of the neuroprotective metabolite kynurenic acid [261]. Likewise, a recent interventional randomized clinical trial has proven the benefits of using flavonoids-enriched orange juice in the alleviation of depressive symptoms, due to an increase in BDNF levels and Lachnospiraceae family in the gut [262]. The combination of polyphenols and other bioactive compounds in coffee, tea, and chocolate also show a promising role in the modulation of gut microbiota and MGB axis [260], thereby supporting the promising role of increasing polyphenols consumption as powerful antidepressant substances.

#### 4.2.2. Lifestyle Intervention

##### Diet

In the above section of prebiotics, we already alluded to advances in the food industry with fortified food in prebiotics and probiotics, especially in fermented food. Here we focus on non-processed and natural sources to stimulate host bacteria. First of all, it should be noted that diet is the greatest gut microbiota shaping factor, therefore improper dietary habits for the individual will determine the dysbiosis status. Unsuitable use of dietary supplements, uncontrolled intermittent fasting, or high fat, animal proteins, and high caloric carbohydrate are some dietary ways to settle the dysbiosis [263].

SCFAs, in their common definition, are metabolic byproducts primarily derived from colon microbial carbohydrate fermentation of dietary fibers that escape digestion in the upper gastrointestinal tract [264]. Therefore, dietary fibers or microbiota-accessible carbohydrates, positively shape the microbial metabolism and the composition of gut microbiota [265,266].

What growing evidence asserts is that well-balanced diets like the Mediterranean Diet, with richness of plant-based ingredients (fruits, vegetables, legumes, olive oil…) fiber, and omega 3 PUFAs, establish a diverse microbiome and produces adequate ratios of imperative microbial metabolites [30]. Here we could mention the vast number of bioactive compounds (polyphenols, carotenoids, phytosterols) that are easy to find in its culinary aromatic spices and herbs (oregano, rosemary, thyme…) conferring health benefits on intestinal barrier and gut microbiota [267,268]. These positive findings have been also associated with the protection against depression and psychosocial maladjustment [265].

With high-fiber diets, *Eubacterium*, *Lactobacillus*, and *Bifidobacterium* species, which are known MAC degraders, are likely to flourish. Short-term (2-week) dietary intervention with MAC showed shifts in bacterial composition, increasing levels of these genera, and upregulated genes involved in inositol degradation [269]. Other studies have demonstrated that, however, not all fermentable fibers can stimulate SCFA production, and they warn about the importance of an individual’s microbial composition when advising certain foods. In this case, *Ruminococcus bromii* and *Clostridium chartatabidum* can contribute to enhanced butyrogenic effects [270]. Recent studies with functional cereals rich in fiber, which can be considered also part of the Mediterranean Diet, test their effectiveness in fecal microbiota composition. For instance, the prebiotic effect of fiber-rich barley increased the population of butyric acid-producing bacteria [271].

Precup and Vodnar [272] revised that due to its metabolite signature, dietary fiber fermenter *Prevotella* is considered a potential biomarker for homeostasis or disease state, nevertheless, in other cases it has been found to be an opportunistic intestinal pathobiont [272] promoting chronic inflammation, mucosal Th17 response and IL-8 and IL-6 production [273]. For this reason, this genus might not be a common probiotic option, because over-representation could lead to low-grade inflammation, so diet intervention with fiber should be oriented at this point to keep the optimal ratio of this genus that contributes to polysaccharide breakdown.

Conversely, many flavonoids from dark chocolate and cocoa powder cause increased neurogenesis and changes in neuronal regions implicated in learning and memory. Their consumption is well known to trigger rapid positive effects on mood even under emotional stress conditions [274]. Theobromine and cacao diets in animal models have shown that SCFA content is higher after intakes, predominantly butyrate production [275]. Similarly, regular tea consumption, due to its richness in teasaponin, catechins, and L-theanine, up-regulates BDNF signaling pathway. These compounds modulate dopaminergic activity and the MGB axis by promoting the growth of *Bifidobacterium* spp. and Lactobacillus/Enterococcus groups, increasing serotonin and GABA [276].

The dietary pattern may also be a major determinant of gut microbial composition and the metabolome. For instance, vegans often present a higher intake of fiber and lower fat in comparison to omnivore individuals that in turn is associated with a reduced level of fecal primary and secondary BAs [277]. It is of note that higher consumption of some animal products (meat, fish, margarine) and fat like fried potatoes were more prominently related to higher BA levels, as well as coffee consumption. Besides, many of these food groups like meat, fish, animal products, or some vegetables like potatoes are also associated with increased choline consumption and hence, upregulated TMA/TMAO levels [181]. Although the detection of both secondary BA and TMA/TMAO levels may be related to depressive symptoms, reducing the consumption of some of these foods may be counterproductive, as the evidence supports the relevance of some products like fish or coffee as potentially neuroprotective and antidepressant agents [278,279]. Instead, for proper microbiota composition and functioning, independently from a vegan or omnivore dietary pattern, it is by far more important to focus on the quality and the origin of the foods, ensuring a high fiber intake, polyphenols, or high-quality fats while limiting overconsumption of red and processed meats, low-quality fats intake or ultra-processed foods) [30].

In general, the depressed patient often adjusts to a profile of malnutrition with a lack of micronutrients we have been mentioning vitamin B, tryptophan. Those are at the same time microbial metabolites, therefore diet ought to supply those deficiencies that are common for gut microbiota and host [280]. Dietary nutrients act as modulators of brain function and behavior, at the same time microbiome metabolism modulates brain function by altering nutrient content from the diet, as precursors for producing its metabolites with psychoactive properties [281]. For the treatment of patients with MDD, the miscellaneous multidimensional microbiota-diet-depression linkage should be addressed [282]. So, we shall not forget Hippocrates’ quote “*let food be thy medicine and medicine be thy food*”.

##### Exercise

Recent studies allege that exercise can enrich microbiota diversity and potentiate beneficial species, which refines microbiota endocrine functions. SCFAs producers may expand by exercise benefits and the MGB axis also becomes modulated [283,284]. Evidence suggests that aerobic exercise triggers diversity and abundance of genera from the Firmicutes phylum, with possible linkage with effects of exercise on the gut and brain [107]. What is undeniable is that synergic effects of microbiota with exercise on cognition and emotion play crucial roles in serotonin signaling and metabolism in general [285].

The antidepressant-like effects of exercise have been compared to drug therapy with fluoxetine in depressed rats, demonstrating that regular exercise treatment could displace bacterial groups: increase Bacteroides, Actinomycetes, and Proteobacteria [286].

The effects of acute moderate-intensity physical activities in amateur athletes display changes in serum and fecal metabolome besides gut microbiota, concrete changes at phenylalanine, tyrosine, and Trp pathways, and a greater abundance of *Rombustia* and *Ruminococcocaceae* genera, which are Trp producers [287].

The already mentioned lactate molecule is another microbial and host metabolite, an important fuel for many cells including neurons and exercise supposes a vital source. The subjacent mechanisms involving this molecule in neuroprotection and memory formation remain unclear [288] but what is known about this compound is to be responsible for mediating exercise-induced adaptations as an ergogenic aid and even being implicated in norepinephrine release and brain plasticity [289]. Additionally, lactate produced during exercise can be utilized by the *Veillonella* genus and trigger its growth. The conversion of lactate to propionate by *Veillonella atypica* has been also positively correlated with run time athletic performance [290].

In athletes’ studies, it is no less important to mention that excess exercise can lead to gastrointestinal problems and psychological conditions like depression, but this training goes frequently hand in hand with improper and troubling dietary recommendations characterized by low consumption of plant polysaccharides, which is associated with reduced microbiota diversity and functionality, less synthesis of SCFAs and neurotransmitters [291].

The already mentioned NaBut can boost their effects thanks to exercising. In a mice model, there were found increased BDNF upregulation and so cognitive benefits of NaBut when administered post-training. Both exercise and NaBut could exert similar epigenetic control mechanisms [292].

The inclusion of exercise, recommendations are encouraging for the patient in terms of stress management besides being a safe, reliable, cost-effective, and pleiotropic effective factor implying multi-organ systemic adaptations [293].

##### Sleep

Currently, it is known that gut microbiota and its metabolites have oscillatory nature, diurnal rhythmicity that responds to the feeding/fasting cycle and that controls sleep duration in the host. The awareness of sleep hygiene is being now more appreciated as interruptions or short sleep durations are associated with hyperactivation of the HPA axis and dysbiosis [294]. Expanding research is demonstrating the nexus between the high incidence of insomnia and MDD with biological rhythms, immune function, and nutrient metabolism. In other words, host sleep and their emotions will depend on circadian rhythms affecting the microbiome [295].

Sleep efficiency has recently been positively correlated with richness in Bacteroidetes and Firmicutes phyla; and these parameters have been linked to the host immune system and cognition [296]. In depressed patients, there are also repeatedly observed shifts in the relative abundance of *Dorea* spp. [218]. This genus has been observed to have the particularity of being related to the quality of sleep simultaneously to depression severity [297]. Then *Dorea* (*Dorea longicatena* and *Dorea formicigenerans*) may be another pathological biomarker closely related to circadian disruption and associated gut microbiota alteration [298] we frequently find in patients with MDD [299,300]. Some advice for restoration of chronobiology in depressed patients could be encouraging them to practice some physical activity or trying to set schedules of eating, working, and then sleeping, aiding to ameliorate the bacterial activity that depends on sleep/wake cycle circadian rhythms. This is also worthy for prevention, as epidemiological studies allege that insomnia in nondepressed individuals is a risk factor for later MDD onset [299].

## 5. Conclusions

**Figure 2:**
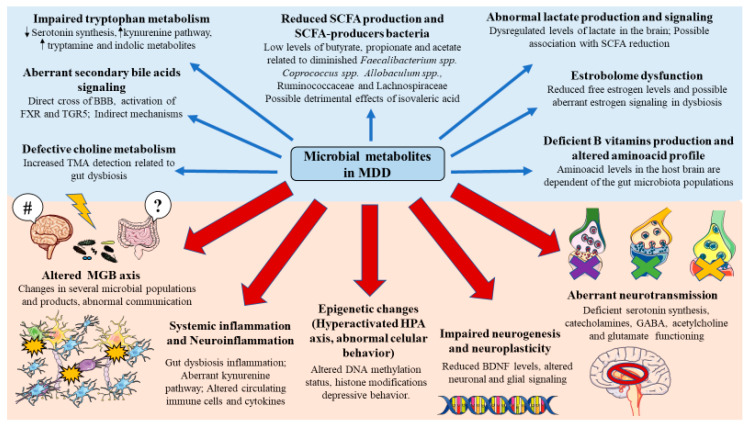
Dysregulation of microbial metabolites in MDD and its consequences. As summarized, a noteworthy dysregulation of SCFAs, lactate, secondary bile acids, choline metabolites, vitamins, amino acids, estrogen, and tryptophan metabolites are potentially involved in the pathogenesis of MDD. Some of the mechanisms reviewed in this article included an altered MGB axis, systemic and local neuroinflammation, epigenetic changes affecting multiple brain regions, impaired neurogenesis and neuroplasticity as well as leading to aberrant neurotransmission.

A deep understanding of metabolic dynamics in gut microbiota is providing another branch in the complex network of MDD modeling (Figure 2). All this knowledge we have witnessed along the last two decades is shedding light on optimizing clinical practice guidelines for diagnosis, treatment, and prevention by integrating more elements to the equation of depressed patient management. Holistic approaches are needed for a successful response to treatment, and this may entail the addition of psychotherapy, antidepressant drugs, and adjuvant food supplements plus the intervention in environmental factors like diet, physical activity, and sleep. Collectively, the influence of these factors would modulate microbially produced metabolites and consequently, promote a more desired activation of host gene expression in homeostatic mechanisms. For greater coverage, the adjuvant strategies that accompany the main treatment, are pursuing to rely on new probiotics (psychobiotics) or pharmabiotics with SCFAs and neuroactive metabolites- producing bacteria that antagonize characteristic depressive stress in the whole MGB axis. Further research and evidence with a critical focus on clinical trials regarding standardized combinations of therapeutic agents with dietary and exercise intervention remains required to support these integrative therapies that exert synergic effects that can better benefit the patient. The main results reviewed in the present work are summarized in Table 1.

## Figures and Tables

**Table 1:** Summarized results reviewed in this work.

Microbial Metabolite	Microorganisms Implicated	Microbiota-Gut-Brain Axis	Status In MDD	Translational Approaches	References
**Short-chain fatty acids (SCFAs)**	*Eubacterium rectale*, *Roseburia faecis*, *Eubacterium hallii*, and *Faecalibacterium prausnitziiappears* to be the major producers of butyrate in the gut. *Veilonella* spp., *Lactobacillus* spp., *Bacteroides* spp., *and Propionibacterium* spp. are some of the most representative bacteria involved in propionate production. Conversely, acetate production is commonly spread among numerous bacterial classes	SCFAs are shown to be a central mechanism of gut-brain communication. They can cross the BBB activating several mechanisms in the brain, modulating the levels of neurotrophic factors, neurotransmitters, neurogenesis, and reducing neuroinflammation and glial dysfunction. SCFAs interact with enteroendocrine cells and promote indirect signaling to the brain by inducing the production of several hormones and neurotransmitters like GABA and serotonin in the gut. There is a positive association between Allobaculum, acetate, and serotonin levels.	Acetate, propionate, butyrate: Downregulated Isovalerate: Upregulated Reduced levels of butyrate producers like Faecalibacterium and *Coprococcus* spp. are correlated with MDD severity. Fecal measures of SCFAs also found a significant association between acetate, propionate, and butyrate levels with both depressive and gastrointestinal symptoms in young adults, therefore supporting that SCFAs are importantly related to the development of MDD. A significant reduction of fecal SCFAs between depressed and non-depressed women was also proposed to be a potential contributor of suffering from MDD with a possible inverse association with depression severity. Depressed patients show a prominent epigenetic footprint in comparison to non-depressed subjects, including critical alterations in DNA methylation, histone modifications, and non-coding RNAs (i.e., microRNAs). SCFAs act as histone deacetylases (HDAC) inhibitors altering gene expression. Propionate production is associated with a decreased anticipatory response of the reward system, related to the monoamine dopamine. Altered dopaminergic neurotransmission is a major feature underpinning MDD, which is related to anhedonia.	SCFA-mediated inhibition of HDAC may lead to the hyperacetylation of histones H3/H4 and hence increase BDNF expression, showing antidepressant effects in mice. This epigenetic modulation is mostly responsible for the immunomodulatory action of SCFAs. In the CNS, SCFAs are major mediators of microglial maturation and function whereas, in the gut, SCFAs induce the differentiation of Treg cells. Overall, the epigenetic and immunomodulatory effects of acetate, and more prominently propionate and butyrate exert promising antidepressant effects, as they are mostly correlated with favorable microbial populations. Prior studies have proven the anti-depressants effect of butyrate by reversing behavioral alterations in mouse models like anhedonia, low energy, cognitive and social abilities. Similarly, the use of rifaximin was related to a decreased depressive-like behavior due to its favorable effects on Ruminococcaceae and Lachnospiraceae, which positively correlated with butyrate production. Conversely, another type of SCFA, isovaleric acid has been shown to be correlated with certain bacterial populations related to MDD and augmented cortisol levels. A proposed mechanism of this pro-depressive effect of isovaleric acid is through interfering with synaptic neurotransmitter release.	[68,69,70,76,77,78,79]
**Lactate**	Lactic acid bacteria, bifidobacteria, or proteobacteria.	Lactate can cross the BBB to match the energetic needs of the brain, influencing many neuronal functions such as excitability, plasticity, and memory consolidation.	The role of lactate in the etiopathogenesis of MDD is poorly understood. However, a plausible explanation is an accumulation of lactate in the brain due to an impaired mitochondrial function -a major feature of different psychiatric diseases-. In MDD, there is increased ventricular lactate. Patients with severe depression show a significant detection of lactate in the urine in comparison to moderate and non-depressed subjects.	Peripheral administration of lactate reverts the effects of stress and exerts antidepressant effects, supporting hippocampal neurogenesis related to changes in serotonin receptor trafficking. Physical exercise may be a powerful link between lactate and antidepressant effects influencing not only the muscle production of lactate but also the MGB axis.	[97,98,99,100,101,102,103,104,105,106,107]
**Tryptophan (Trp) metabolites**	Some bacterial communities may synthesize Trp like *Escherichia coli*. 5 phyla are involved in Trp metabolism: Firmicutes, Bacteroidetes, Actinobacteria, Proteobacteria, and Fusobacteria, with a central role of Clostridium, Burkholderia, Streptomyces, Pseudomonas, and Bacillus genera	Trp is importantly implicated in the synthesis of serotonin and other critical metabolites, including those belonging to the kynurenine pathway; tryptamine; indole, and derivates. All these components exert signaling actions in the brain, involved in its proper functioning.	Serotonin synthesis from Trp is impaired An altered kynurenine pathway is reported in patients with MDD, with an upregulation of the enzyme indoleamine 2 3-dioxygenase (IDO) and neurotoxic components like 3-hydroxykynurenine and quinolinic acid along with a reduction of neuroprotective members like kynurenine and kynurenic acid Increased tryptamine levels and indole metabolism	Probiotic bacteria (*Bifidobacterium longum* subsp. infantis E41 and *Bifidobacterium breve* M2CF22M7) were associated with an increased 5-hydroxytryptophan, tph1, serotonin, and BDNF in the brain. Current clinical trials are evaluating the efficacy of different probiotic strains with prebiotics and antidepressants in the Trp metabolism, kynurenine pathway, and other pathophysiological mechanisms involved in MDD. Anthocyanins, a group of polyphenols used as nutraceutical appears to modulate host Trp metabolism increasing neuroprotective kynurenic acid Ensuring an adequate nutritional and dietary context is crucial to prevent gut dysbiosis and Trp metabolism, although the evidence has failed to find benefits from increasing dietary Trp or through supplementation	[30,112,113,114,115,116,117,119,120,121,125,126,127,128,129,261]
**Amino acids**	Many bacterial groups are involved in the synthesis and metabolism of several amino acids including glutamate, phenylalanine, tyrosine, threonine, isoleucine, alanine, serine, and oxidized proline	Glutamate is an important neurotransmitter as well as GABA, which is synthesized from glutamate by different microbial communities, especially lactic acid bacteria. Phenylalanine is involved in the synthesis of the catecholamines epinephrine, norepinephrine, and dopamine	Glutamate, GABA, and catecholamines functioning in the brain is impaired in patients with MDD Chronic unpredictable mild stress rats, as well as specific pathogen-free (SPF) and germ-free (GF), have unraveled that gut microbiota is critical for serum levels of plenty amino acid, as well as the concentration of different amino acids in the host brain regions Reduced levels of phenylalanine, aspartate, glutamate, and serine are found in patients with MDD and heart failure	SCFAs from vinegar (acetic acid) is associated with an improved amino acid metabolism (Increased levels of glycine, serine, and threonine) in healthy subjects Creatine supplementation (Nutraceutical) may be related to an improved amino acid metabolism Little and controversial evidence is available regarding the benefits from the administration of some amino acids like phenylalanine and tyrosine Targeting gut microbiota and gut dysbiosis through lifestyle interventions seems to be the most adequate strategy to influence amino acid levels and metabolism	[131,132,133,134,136,137,138,139,140,141,143,144,145,247,251]
**Secondary bile acids (BAs)**	Clostridium, Enterococcus, Bifidobacterium, Lactobacillus, Bacteroides genera, as well as *E. coli*, *C. testosteroni*, and *Ruminococcus* spp. Archaea like *Methanobrevibacter smithii* and *Methanosphera stadmanae*	BAs exert important signaling actions in the brain through Farnesoid-X-Receptor (FXR) and the G-Protein-Coupled Bile Acid receptor-1 (GPBAR-1). BAs may act directly by crossing the blood–brain barrier or indirectly, activating FXR and GPBAR-1 receptors in the gut, leading to the production of fibroblast growth factor 19 (FGF19) and glucagon-like peptide 1 (GLP-1), signaling to the brain	In neuropsychiatric disorders, the most prominent signaling route of BA is through the FXR. In the hippocampus, an increased FXR expression seems to cause depression-like symptoms and reduced BDNF levels in rats The use of some antidepressants such as paroxetine alter gut microbiota and BA production in mice	The activation of GPBAR-1 by the secondary bile acid tauroursodeoxycholic acid (TUDCA) as well as the activation of other receptors in the brain of BA like vitamin D receptor (VDR) and pregnane X receptor (PXR) activated by lithocholic acid (LCA) appears to present antidepressant effects Higher consumption of animal products and fats is associated with increased BA production, which is in turn inversely correlated with dietary fiber intake. Ensuring an adequate fiber intake, polyphenols, and high-quality fats while limiting overconsumption of red and processed meats, low-quality fats intake, or ultra-processed foods will benefit BA metabolism and actions in the brain	[30,149,150,152,153,157,158,159,160,277]
**Vitamins**	B1 producers are *Bacteroides fragilis* and *Prevotella copri* (phylum Bacteroidetes); *Clostridium difficile*, some *Lactobacillus* spp., and *Ruminococcus lactaris* (Firmicutes); *Bifidobacterium* spp. (Actinobacteria); and *Fusobacterium varium.* *Bacteroides fragilis* and *Prevotella copri* (Bacteroidetes); *Clostridium difficile*, *Lactobacillus plantarum*, *L. fermentum*, and *Ruminococcus lactaris* (Firmicutes) can synthesize B2 vitamin. *Bacteroides fragilis* and *Prevotella copri* (Bacteroidetes); *Ruminococcus lactaris*, *Clostridium difficile* (Firmicutes); *Bifidobacterium infantis* (Actinobacteria); *Helicobacter pylori* (Proteobacteria); and *Fusobacterium varium* (Fusobacteria) can produce B3 vitamin. B3 can be synthesized from Trp as well. *Bacteroides fragilis* and *Prevotella copri* (Bacteroidetes); some *Ruminococcus* spp. (R. *lactaris* and R. *torques*) (Firmicutes); *Salmonella enterica* and *Helicobacter pylori* (Proteobacteria) synthesize B5. B6 producers are *Bacteroides fragilis* and *Prevotella copri* (Bacteroidetes), *Bifidobacterium longum* and, *Collinsella aerofaciens* (Actinobacteria), and *Helicobacter pylori* (Proteobacteria). B7 producers are *Bacteroides fragilis* and *Prevotella copri* (Bacteroidetes); *Fusobacterium varium* (Fusobacteria) and *Campylobacter coli* (Proteobacteria) Folate or B9 is produced by *Bacteroides fragilis* and *Prevotella copri* (Bacteroidetes); *Clostridium difficile*, *Lactobacillus plantarum*, *L. reuteri*, *L. delbrueckii* ssp. *bulgaricus*, and *Streptococcus thermophilus* (Firmicutes), some species in *Bifidobacterium* spp. (Actinobacteria); *Fusobacterium varium* (Fusobacteria) and *Salmonella enterica* (Proteobacteria). Finally, B12 is produced by *Bacteroides fragilis* and *Prevotella copri* (Bacteroidetes); *Clostridium difficile*, *Faecalibacterium prausnitzii* and *Ruminococcus lactaris* (Firmicutes); *Bifidobacterium animalis*, *B.infantis*, and *B.longum* (Actinobacteria); *Fusobacterium varium* (Fusobacteria). *Lactobacillus plantarum* and *L. coryniformis* from fermented food also produce B12.	Gut dysbiosis impairs the synthesis of these vitamins which are crucial for the proper functioning of the brain.	Patients with MDD show deficiencies of vitamins, especially D and those from B complex. Low levels of vitamin B1 (thiamine), B2 (riboflavin) B3 (niacin), B6 (pyridoxine) B7/8 (biotin), B9 (folate), and B12 (cyanocobalamin) are pivotal B vitamins associated with MDD.	Besides vitamin D supplementation improves gut microbiota in MDD, several studies are confirming the benefits from using certain B vitamins in combination with probiotics, frequently represented by different species and strains of Bifidobacterium and Lactobacillus. In some studies, in comparison to the groups that only received vitamin B7 plus placebo or probiotics plus placebo, patients supplemented with both probiotics and vitamin showed an increased synthesis of vitamin B6 and B7 and improved clinical outcomes. Gut microbiota is crucial for determining the body levels of B vitamins, but an adequate dietary context is needed to obtain the benefits from those bacteria involved in vitamin synthesis, with noteworthy positive outcomes for the brain.	[164,165,166,167,168,169,301]
**Choline-derived metabolites**	Firmicutes phyla (Clostridium cluster XIVa and Eubacterium strains), Actinobacteria, and Proteobacteria	Choline may be transformed into plenty of metabolites like trimethylamine (TMA), betaine, phosphocholine, and acetylcholine (neurotransmitter). The gut microbiota status and the TMA production and its metabolic product derived, trimethylamine N-oxide (TMAO) are crucial biomarkers of choline metabolism and predictors of the beneficial or detrimental effects in the brain	An increased TMA and TMAO levels related to gut dysbiosis are directly correlated depressive symptoms severity in females and males Elevated levels of choline-consuming bacteria are related to an aberrant global DNA methylation pattern in mice and abnormal behavior in mice	Similar to BA, some animal products and fatty foods may contain high levels of choline and then, it may raise TMA/TMAO levels. However, if maintaining a eubiotic environment with an adequate dietary fiber intake, polyphenols, vitamins, high-quality fats while limiting overconsumption of red and processed meats, low-quality fats, excessive salt, additives, or ultra-processed foods TMA levels will not have any detrimental effect. Conversely, the variety of components included in a proper dietary pattern like those included in the Mediterranean diet will benefit the host health and aid to ameliorate depressive symptoms.	[30,170,171,176,177,179,180,181,182,183]
**Estrobolome**	This term refers to a set of bacteria that are able of metabolizing and modulate the estrogen levels in the body. The gut microbiota is a major determinant of the circulating levels of estrogens through different mechanisms. In men and postmenopausal women, gut microbiota diversity and functions influence the levels of non-ovarian estrogens via enterohepatic circulation, where Clostridia taxa and genera from the Ruminococcaceae family could be key microbial populations involved in this regulation.	Gut microbiota may influence estrogen metabolism and signal in the brain. There are some neuroprotective effects of estrogens mediating some neuroplastic actions in the hippocampus and regulating serotonin receptors levels.	Gut dysbiosis may significantly impair the action of gut microbiota in the estrogen metabolism, driving to the reduction of circulating estrogens, which is associated with a plethora of adverse outcomes ranging from obesity, metabolic syndrome, malignancies, endometrial hyperplasia, endometriosis, polycystic ovary syndrome, fertility, cardiovascular disease, and cognitive dysfunction. The evidence supporting the role of altered sexual hormones in the pathogenesis of MDD in women could be found in the existence of three different types of depression related to the menstrual cycle: Premenstrual dysphoric disorder (PMDD), postpartum depression, and perimenopausal depression. The use of estradiol, but not progesterone appears to be effective for PMDD, postpartum, and moderate/severe perimenopausal depression.	Despite neuroprotective effects, the use of estradiol as the first line-treatment is not supported by current scientific evidence Instead, targeting the estrobolome in the prevention and treatment of these specific disorders could be an effective interesting approach, as women may benefit from the actions of these bacteria. Some studies have reported that lower levels of testosterone but estrogen were associated with more severe depressive symptoms in a group of women with MDD. Other reports allege that gut microbiota transplantation of male to female mice led to an augmentation of testosterone levels so future studies may consider the benefits from using testosterone-producer microorganisms to raise testosterone levels. As gut microbial diversity is directly related to the levels of non-ovaric estrogen levels and the intake of certain dietary components like polyphenols may aid in the modulation of beneficial microbial communities and ER activation, nutritional and lifestyle interventions may be a potential strategy for boosting the estrobolome.	[184,188,189,190,191,192,193,194,195]
